# Concurrent Primary Repair of a Glenoid Labrum Articular Disruption and a Bankart Lesion in an Adolescent: A Case Report of a Novel Technique

**DOI:** 10.1155/2019/4371860

**Published:** 2019-02-10

**Authors:** Avinesh Agarwalla, Richard N. Puzzitiello, Natalie L. Leong, Brian Forsythe

**Affiliations:** ^1^Midwest Orthopaedics at Rush, Rush University Medical Center, Chicago, IL, USA; ^2^Department of Orthopaedic Surgery, University of Maryland, College Park, MD, USA

## Abstract

Glenoid labrum articular disruption (GLAD) lesions are an uncommon concomitant injury associated with labral tears, occurring in 1.5-2.9% of cases. In previous reported cases, the articular lesion is debrided during repair of the labral injury, which may predispose patients to osteoarthritis, a longitudinal complication seen in articular debridement of the hip. We report the case of a 15-year-old healthy adolescent male swimmer who sustained a labral injury with a concomitant GLAD lesion. During operative management, three Polyetheretherketone (PEEK) SutureTaks were placed on the glenoid. #2 FiberWire was used to imbricate capsular tissue, passed beneath the labrum, and was then subsequently advanced through the fibrous rim of the displaced cartilage flap/GLAD lesion at the site of each suture anchor. This construct restored tension to the anterior band of the inferior glenohumeral ligament, recreated the anteroinferior labral bumper, and effectively reduced the cartilage flap/GLAD lesion to the anterior inferior glenoid. By six months postoperatively, the patient demonstrated near-normal function with full range of motion and evidence of a stable construct on MRI. Unlike previously described cases, this is the first report of a hybrid technique that simultaneously performed a primary repair of both labral and articular injuries without the use of additional implants for the articular lesion. Primary repair of the labral and articular lesions should provide longitudinal benefit to the patient by reducing the risk of developing glenohumeral osteoarthritis.

## 1. Introduction

Anterior inferior glenolabral injuries occur secondary to anterior shoulder dislocations and are often associated with concomitant injuries such as the Bankart lesion, anterior labral periosteal sleeve avulsions (ALPSA), Perthes lesions, and glenoid labral articular disruption (GLAD) lesions [1]. GLAD lesions are the least common of these concomitant injuries, occurring in 1.5-2.9% of anterior inferior glenolabral injuries. This injury most commonly occurs with forced adduction while the arm is abducted and externally rotated and is often a significant source of anterior shoulder pain in these patients [1–3].

GLAD lesions are characterized by an avulsion of the articular glenoid cartilage that is associated with the inferior glenoid labrum, due to the continuity of the deep fibers of these structures [2, 4]. A high degree of clinical suspicion is needed due to the infrequency of these injuries. Furthermore, GLAD lesions are not readily visualized on standard MRI [5]; thus, higher resolution studies such as magnetic resonance arthrography (MRA) may be needed to detect this injury pattern [1, 3, 5]. Management of GLAD lesions involves arthroscopic debridement or chondroplasty to correct the uneven chondral layer that may be the source of discomfort, in addition to a stabilization procedure for concurrent labral injury [1, 2, 6].

In this case report, we present an adolescent male with a previous history of arthroscopic shoulder surgery who experienced a shoulder dislocation while swimming. The patient was diagnosed with an anterior inferior glenolabral tear with a GLAD defect and underwent primary arthroscopic labral repair with concomitant glenolabral cartilage disruption repair, utilizing a previously unreported single suture anchor construct.

## 2. Case Report

The patient is a 15-year-old competitive male swimmer with a history of bilateral arthroscopic subacromial decompression within the preceding year. The patient continued regular follow-up with the senior author until he reached maximal medical improvement (MMI) from these procedures. One week following this visit, the patient suffered a right shoulder dislocation while swimming, which was self-reduced. He presented to the clinic 3 days following the injury. At this time, he reported mild pain (3/10), and his self-reported functionality was less than 20% of normal.

Upon presentation, the patient was not in acute distress, and there was no obvious deformity of the right shoulder. He reported tenderness to palpation on the bicipital groove and achieved 150 degrees of scaption, 45 degrees of external rotation, and internal rotation to the T10 level. He demonstrated a positive Neer test, Hawkins test, O'Brien's test, and valgus sheer test. He demonstrated a positive anterior load test. He demonstrated a negative posterior load test, belly test, and a lift-off test. An MRI was ordered to evaluate his labrum, which demonstrated a humeral head subluxation with posterior humeral head contusion and Buford complex. Conservative management with physical therapy was recommended at this time.

After six weeks of physical therapy, the patient returned for evaluation and noted moderate pain (4/10), function less than 50% of normal, and instability. He was experiencing serious discomfort using a ladder and experienced an episode of shoulder subluxation. His physical examination findings were largely unchanged from his previous visit but exhibited discomfort with apprehension and anterior load examinations.

**Figure 1:**
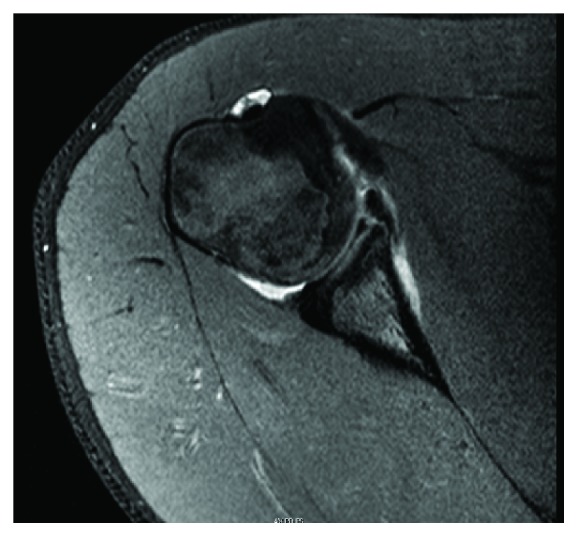
Preoperative MRI that was initially read as a Buford complex, but after failed conservative management, the lesion was reevaluated as an anterior inferior labral tear.

Following examination, his previous MRI was again reviewed. While the official report described a Buford complex, the abnormal-appearing labrum was located more inferior than the typical Buford complex—consistent with an anterior labral tear (Figure 1). Given his inability to return to sport activities and MRI results consistent with a labral injury, it was recommended that he undergo arthroscopic anterior labral repair due to his lack of progress from conservative management. The patient and his family elected to proceed with operative management.

**Figure 2:**
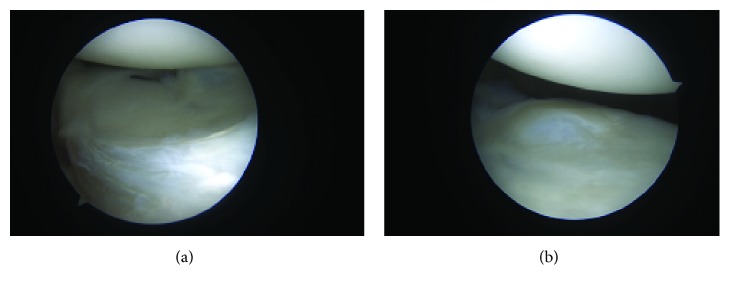
Anterior inferior labral injury at the 3 o'clock (a) and 6 o'clock (b) positions.

**Figure 3:**
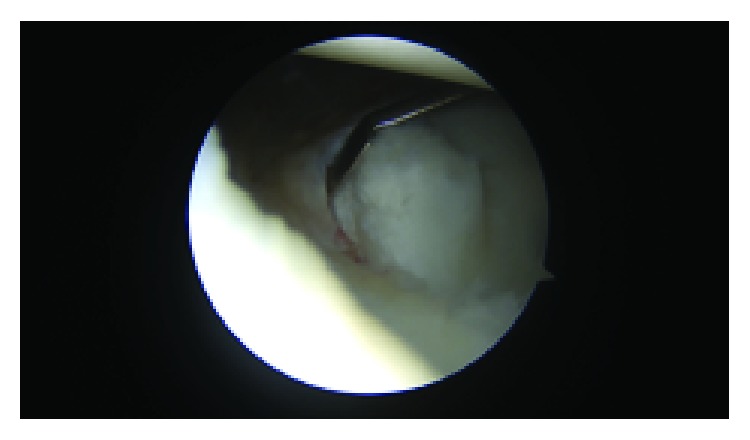
Identification of a 6 mm × 8 mm glenolabral articular defect (GLAD).

During the procedure, the patient was placed in the lateral decubitus position. Standard anterior and posterior portals were established with an accessory anterior superolateral viewing portal. Diagnostic arthroscopy was significant for complete disruption of the labral tissue between the 3 o'clock and 6 o'clock positions (Figures 2(a) and 2(b), respectively). It was also revealed that there was an articular glenoid cartilage lesion measuring 6 mm × 8 mm anteroinferiorly (Figure 3). The posterior and superior labrums were intact, and there was no damage to any rotator cuff tendon or the biceps tendon.

**Figure 4:**
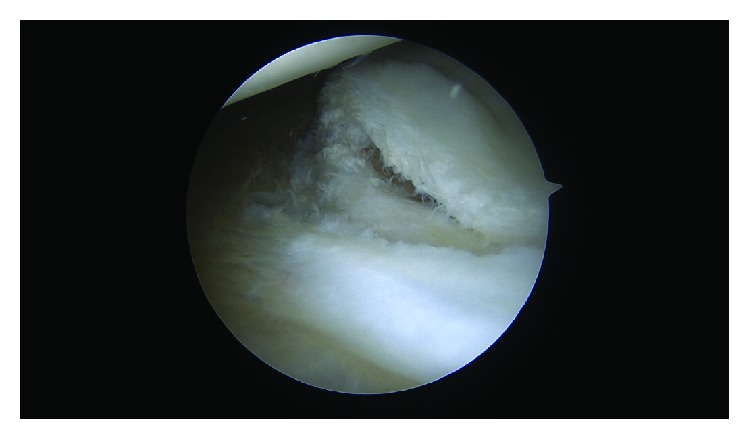
GLAD lesion following arthroscopic debridement of the underlying bony surface.

**Figure 5:**
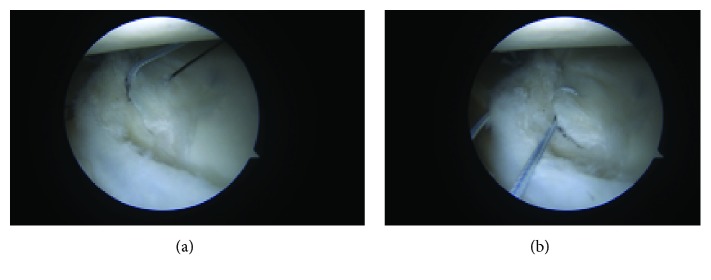
Primary repair of the GLAD lesion. #2 suture is passed through the cartilage (a) with the fixation being incorporated into anchor constructs of the labral repair.

**Figure 6:**
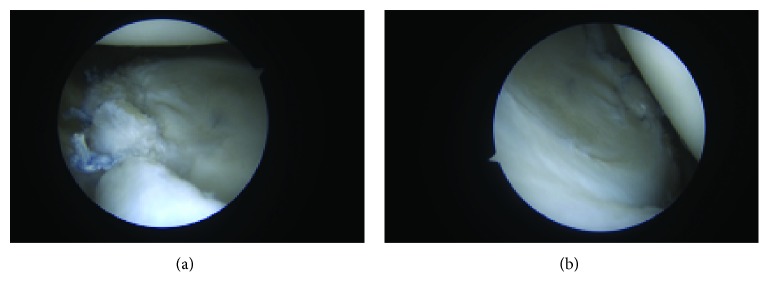
Final repair of the anterior inferior labrum and GLAD lesion.

Before the labral repair, the calcified cartilage layer along the anterior-inferior glenoid, beneath the cartilage flap, was gently debrided and removed with an arthroscopic shaver and an arthroscopic curette (Figure 4). An arthroscopic biter and shaver were utilized to trim the fibrillated margins of the cartilage tissue. Three 2.4 mm Polyetheretherketone (PEEK) (Arthrex, Naples, FL) SutureTaks were placed at the 6, 4:30, and 3 o'clock positions on the glenoid. A 25-degree right angle suture lasso (Arthrex, Naples, FL) was used to imbricate several millimeters of capsular tissue. The suture lasso was then passed beneath the labrum at the site of each suture anchor and was then subsequently advanced through the fibrous rim of the displaced cartilage flap/GLAD lesion. The Nitinol wire was used to shuttle #2 FiberWire though the cartilage flap, around the labrum, and through the capsular tissues to establish 3 fixation points (Figures 5(a) and 5(b)). A simple knot configuration was tied with sliding arthroscopic Weston knots followed by three alternating half stitches, repeated 3 times, beginning inferiorly at 6 o'clock, progressing superiorly to 4:30 and 3 o'clock. This construct restored tension to the anterior band of the inferior glenohumeral ligament, recreated the anteroinferior labral bumper, and effectively reduced the cartilage flap/GLAD lesion to the anterior inferior glenoid (Figures 6(a) and 6(b)).

Three months postoperative, the patient reported no pain and 85% of normal function. On physical examination, he achieved 165 degrees of scaption, 65 degrees of external rotation, and internal rotation to the level of T9. At this time, he was instructed to continue physical therapy as per standard institutional protocol.

**Figure 7:**
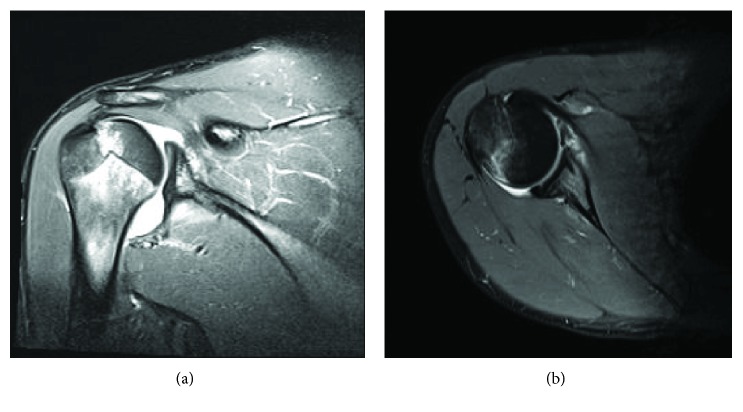
Coronal (a) and axial (b) MRI performed 5 months postoperative due to traumatic injury reveals a Hill-sachs lesion. After nonconservative treatment resulted in improvement, the patient was instructed to follow-up only as needed.

Five months postoperative, the patient suffered a traumatic fall backwards and landed on his outstretched, extended hand. He felt that his shoulder may have subluxed and noted moderate pain with activity (5/10). On physical examination, he achieved 150 degrees of scaption with discomfort, 45 degrees of external rotation with discomfort, and internal rotation to the level of T10. He had discomfort with apprehension, which was relieved with relocation, as well as discomfort with anterior load and shift. An MRI was performed which revealed an intact GLAD lesion repair and labral repair (Figures 7(a) and 7(b), respectively). The GLAD lesion repair remained intact. Given the acuity of the injury, it was recommended that he undergo conservative treatment for one month.

The patient was seen one month later at his 6-month postoperative visit and reported no pain and 95% normal function. On physical examination, he achieved 170 degrees of scaption, 50 degrees of external rotation, and internal rotation to the level of T5 without any discomfort. He exhibited no tenderness to palpation and demonstrated a negative Neer test, O'Brien's test, valgus sheer stress, belly press test, anterior load test, and posterior load test. The apprehension test was similarly negative. The patient was instructed to continue with his home exercise regimen.

## 3. Discussion

Management of glenoid labral articular disruption (GLAD) is predicated on creating an even articular surface by debriding chondral tissue and removing loose bodies from the glenohumeral joint space [1, 2, 6]. The course of operative management is often determined by the degree of the articular lesion. If a small lesion is encountered, the unstable articular flap can be debrided, and the labrum can be advanced into the defect; however, if there is diffuse damage, only the articular surface is debrided, and the defect is left unfilled [7]. In this case report, we present an adolescent male who suffered an anterior shoulder dislocation which caused an anterior inferior labral tear with a concomitant GLAD lesion, which were both arthroscopically repaired concurrently with a single suture anchor construct. We believe that our technique is novel and previously unreported.

Traditionally, glenoid labral articular defects are repaired separately to labral lesions. Galano et al. utilized a cartilage fixation device to repair the GLAD lesion followed by labral repair with multiple suture anchors [8]. Additionally, Page and Bhatia reported a technique that utilized multiple suture anchors to first imbricate labral tissue and fasten it to the glenoid bone [9]. During fixation, the labral tissue was overlapped with the chondral edge to provide stability to the articular lesion, while additional anchoring stitches were then implanted to further secure the articular tissue. While primary repair of the GLAD lesion was described, none of these aforementioned techniques utilized a single suture anchor construct to concurrently repair both chondral and labral lesions.

Several studies have shown radiographic and clinical evidences demonstrating that patients receiving knee meniscectomies are at an increased risk of developing osteoarthritis due to loss of cartilage [10–12]. While studies have yet to establish a relationship between articular damage and the development of glenohumeral osteoarthritis, the same principles of increased humeral-to-glenoid contact following cartilage deficit lead to bony lesions within the glenohumeral joint. In older patients with glenohumeral osteoarthritis, surgical management typically includes arthroscopic debridement in mild cases, and in refractory or severe cases, a total shoulder arthroplasty [13, 14]. In younger patients, the treatment algorithm is not as clearly elucidated since radiologic and clinical outcomes following intervention are less favorable than in older patients [15].

Although the complications and outcomes of labral injuries with concomitant articular damage in the shoulder have not been thoroughly determined, within the hip literature, labral tears are frequently associated with articular lesions in the anterior acetabulum [16]. This injury predisposes patients to hip instability and osteoarthritis secondary to the labral defect and articular damage, respectively [17]. Although patients receiving labral repairs have favorable outcomes, those with concomitant cartilage defect are at an increased risk of osteoarthritis due to increased contact pressure of the femoral head on the acetabulum [18, 19]. Due to the analogous anatomy and physiology between the structures of the shoulder and hip joints, labral injuries with concomitant cartilage defect in the shoulder may lead to the development of osteoarthritis over time.

While concomitant injuries and fixation techniques impact the outcomes of shoulder labral repair in adolescents, patients exhibit good to excellent outcomes following arthroscopic repair [20]. The recurrence rate of instability following operative management is higher among the adolescent population than adults (18.7% vs. 25.1%, respectively); however, nearly 82% of adolescent patients return to their preinjury sport [21–23]. Due to the infrequent nature of GLAD lesions, outcomes following this injury have not been reported. Gross et al. performed a systematic review on cartilage restorative and reparative procedures of the glenohumeral joint, and while each injury and corrective procedure reported favorable outcomes, sample heterogeneity and a small sample size prohibited a direct comparison [24].

Management of GLAD lesions has been previously described with the purpose of reducing the uneven chondral surface within the glenohumeral joint in order to reduce pain [1, 2]. However, chondral debridement may predispose to glenohumeral arthritis. In this report, a patient who suffered an anterior inferior labral tear with concomitant GLAD lesion underwent concurrent primary repair of both the GLAD lesion and labral tear, utilizing a single suture anchor construct. Nonabsorbable sutures were used instead of absorbable sutures because they provide a more durable fixation during the healing phase (first four weeks postoperative). The simple stitch configuration is of a low profile; thus, no suture knot crosses the articular surface in this repair construct. If the GLAD lesion was managed with articular debridement, exposure of the underlying glenoid bone may have eventually predisposed to discomfort, instability, pain, and osteoarthritis. Glenohumeral arthritis may evolve to a debilitating disability with effects on quality of life comparable to chronic medical conditions such as chronic heart failure or diabetes [25]. Our approach to concurrent anterior labral and GLAD lesion repair should benefit the patient longitudinally by reducing the risk of developing osteoarthritis.

## References

[1] Waldt S., Burkart A., Imhoff A. B., Bruegel M., Rummeny E. J., Woertler K. (2005). Anterior shoulder instability: accuracy of MR arthrography in the classification of anteroinferior labroligamentous injuries. *Radiology*.

[2] Neviaser T. J. (1993). The GLAD lesion: another cause of anterior shoulder pain. *Arthroscopy*.

[3] Antonio G. E., Griffith J. F., Yu A. B., Yung P. S. H., Chan K. M., Ahuja A. T. (2007). First-time shoulder dislocation: high prevalence of labral injury and age-related differences revealed by MR arthrography. *Journal of Magnetic Resonance Imaging*.

[4] Boileau P., Villalba M., Hery J. Y., Balg F., Ahrens P., Neyton L. (2006). Risk factors for recurrence of shoulder instability after arthroscopic Bankart repair. *The Journal of Bone & Joint Surgery*.

[5] Pavic R., Margetic P., Bensic M., Brnadic R. L. (2013). Diagnostic value of US, MR and MR arthrography in shoulder instability. *Injury*.

[6] Page R. S. (2008). Managing chondral lesions of the glenohumeral joint. *International Journal of Shoulder Surgery*.

[7] Elser F., Braun S., Dewing C. B., Millett P. J. (2010). Glenohumeral joint preservation: current options for managing articular cartilage lesions in young, active patients. *Arthroscopy*.

[8] Galano G. J., Weisenthal B. M., Altchek D. W. (2013). Articular shear of the anterior-inferior quadrant of the glenoid: a glenolabral articular disruption lesion variant. *American Journal of Orthopedics*.

[9] Page R., Bhatia D. N. (2010). Arthroscopic repair of a chondrolabral lesion associated with anterior glenohumeral dislocation. *Knee Surgery, Sports Traumatology, Arthroscopy*.

[10] Englund M., Lohmander L. S. (2004). Risk factors for symptomatic knee osteoarthritis fifteen to twenty-two years after meniscectomy. *Arthritis & Rheumatism*.

[11] Roos E. M., Roos H. P., Lohmander L. S., Ekdahl C., Beynnon B. D. (1998). Knee Injury and Osteoarthritis Outcome Score (KOOS**)**—development of a self-administered outcome measure. *Journal of Orthopaedic & Sports Physical Therapy*.

[12] Johnson R. J., Kettelkamp D. B., Clark W., Leaverton P. (1974). Factors effecting late results after meniscectomy. *The Journal of Bone & Joint Surgery*.

[13] Weinstein D. M., Bucchieri J. S., Pollock R. G., Flatow E. L., Bigliani L. U. (2000). Arthroscopic debridement of the shoulder for osteoarthritis. *Arthroscopy*.

[14] Radnay C. S., Setter K. J., Chambers L., Levine W. N., Bigliani L. U., Ahmad C. S. (2007). Total shoulder replacement compared with humeral head replacement for the treatment of primary glenohumeral osteoarthritis: a systematic review. *Journal of Shoulder and Elbow Surgery*.

[15] Denard P. J., Wirth M. A., Orfaly R. M. (2011). Management of glenohumeral arthritis in the young adult. *The Journal of Bone and Joint Surgery-American Volume*.

[16] McCarthy J. C., Noble P. C., Schuck M. R., Wright J., Lee J. (2001). The watershed labral lesion: its relationship to early arthritis of the hip. *The Journal of Arthroplasty*.

[17] McCarthy J. C., Noble P. C., Schuck M. R., Wright J., Lee J. (2001). The role of labral lesions to development of early degenerative hip disease. *Clinical Orthopaedics and Related Research*.

[18] Beck M., Kalhor M., Leunig M., Ganz R. (2005). Hip morphology influences the pattern of damage to the acetabular cartilage: femoroacetabular impingement as a cause of early osteoarthritis of the hip. *The Journal of Bone and Joint Surgery-British Volume*.

[19] Harris J. D. (2016). Hip labral repair: options and outcomes. *Current Reviews in Musculoskeletal Medicine*.

[20] Imhoff A. B., Ansah P., Tischer T. (2010). Arthroscopic repair of anterior-inferior glenohumeral instability using a portal at the 5:30-o'clock position: analysis of the effects of age, fixation method, and concomitant shoulder injury on surgical outcomes. *The American Journal of Sports Medicine*.

[21] Rose G. D., Borroni M., Castagna A. (2013). The role of arthroscopic capsulo-labral repair in unidirectional post-traumatic shoulder instability in adolescent athletes participating in overhead or contact sports. *Joints*.

[22] Deitch J., Mehlman C. T., Foad S. L., Obbehat A., Mallory M. (2003). Traumatic anterior shoulder dislocation in adolescents. *The American Journal of Sports Medicine*.

[23] Mazzocca A. D., Brown F. M., Carreira D. S., Hayden J., Romeo A. A. (2005). Arthroscopic anterior shoulder stabilization of collision and contact athletes. *The American Journal of Sports Medicine*.

[24] Gross C. E., Chalmers P. N., Chahal J. (2012). Operative treatment of chondral defects in the glenohumeral joint. *Arthroscopy*.

[25] Gartsman G. M., Brinker M. R., Khan M., Karahan M. (1998). Self-assessment of general health status in patients with five common shoulder conditions. *Journal of Shoulder and Elbow Surgery*.

